# Acid-Catalysed Conversion of Saccharides into Furanic Aldehydes in the Presence of Three-Dimensional Mesoporous Al-TUD-1

**DOI:** 10.3390/molecules15063863

**Published:** 2010-05-28

**Authors:** Sérgio Lima, Margarida M. Antunes, Auguste Fernandes, Martyn Pillinger, Maria Filipa Ribeiro, Anabela A. Valente

**Affiliations:** 1 Department of Chemistry, CICECO, University of Aveiro, 3810-193 Aveiro, Portugal; E-Mails: sergiolima@ua.pt (S.L.); margarida.antunes@ua.pt (M.M.A.); mpillinger@ua.pt (M.P.); 2 Institute for Biotechnology and Bioengineering, Centre for Biological and Chemical Engineering, Instituto Superior Técnico, Av. Rovisco Pais, 1049-001 Lisboa, Portugal; E-Mails: auguste.fernandes@ist.utl.pt (A.F.); filipa.ribeiro@ist.utl.pt (M.F.R.)

**Keywords:** solid acid, mesoporous materials, biomass valorisation, 2-furfuraldehyde, 5-hydroxymethylfurfural

## Abstract

The one-pot acid-catalysed conversion of mono/di/polysaccharides (inulin, xylan, cellobiose, sucrose, glucose, fructose, xylose) into 2-furfuraldehyde (FUR) or 5-hydroxymethylfurfural (HMF) in the presence of aluminium-containing mesoporous TUD-1 (denoted as Al-TUD-1, Si/Al = 21), at 170 ºC was investigated. Xylose gave 60% FUR yield after 6 h reaction; hexose-based mono/disaccharides gave less than 20% HMF yield; polysaccharides gave less than 20 wt % FUR or HMF yields after 6 h. For four consecutive 6 h batches of the xylose reaction in the presence of Al-TUD-1, the FUR yields achieved were similar, without significant changes in Si/Al ratio.

## 1. Introduction

The conversion of renewable biomass resources into non-petroleum derived fuels and chemicals is becoming increasingly attractive as a way to avoid intensification of global warming and diversify energy sources [1,2,3,4]. Several low-cost kinds of plant biomass may be utilised, such as agricultural and forest waste and surpluses, plants grown on arid land, and aquatic plants. The major components of plant-derived biomass are carbohydrates, which may be converted into a number of valuable platform compounds, such as 2-furfuraldehyde (FUR) and 5-hydroxymethylfurfural (HMF). FUR and HMF may be produced via acid-catalysed hydrolysis of di/oligo/polysaccharides into the respective monosaccharides, followed by cyclodehydration of the latter. FUR is produced on an industrial scale (commonly using H_2_SO_4_ as the catalyst) for a plethora of applications [5,6]. To the best of our knowledge, HMF has not reached industrial scale production. 

Efforts have been made over the past few years to develop stable, recyclable, solid Brønsted and/or Lewis acids based on inorganic oxides for the conversion of saccharides into FUR and HMF [7,8,9,10,11,12,13,14,15]. Of the studied catalysts, microporous zeolites or zeotype materials are quite promising [7,8,9,10,11]. However, the transformation of relatively bulky saccharides may be hindered in a microporous structure, and hence the use of mesoporous aluminosilicates may be preferable [16]. In 1992 Kresge *et al.* reported the synthesis of ordered mesoporous aluminosilicates using surfactants as templates [17]. The so-called M41S materials possess high specific surface areas and pore volumes, and uniform but controllable pore sizes in the range of 2–10 nm, which makes them interesting candidates as catalysts or catalyst supports. Many other types of mesoporous materials have since been described, most of which are prepared using surfactants or polymers as structure-directing agents. Economic concerns have motivated the search for low-cost, non-surfactant templating routes to mesoporous materials. An important discovery was the straightforward synthesis of the three-dimensional sponge-like mesoporous (siliceous) oxide TUD-1, using either triethanolamine or tetraethyleneglycol as organic templates [18]. The purely siliceous TUD-1 may be furnished with Brønsted and Lewis acidity by the incorporation of metals such as Al into the framework via a one-pot procedure based on the sol-gel technique [19,20,21,22,23,24]. The high specific surface area, pore volume and width of TUD-1 and related materials, coupled with the 3-D channel system, may facilitate diffusion and promote the access of bulky reagents to active sites.

In the present work, aluminium-containing mesoporous TUD-1 (denoted as Al-TUD-1) has been investigated for the first time as a solid acid catalyst in the acid-catalysed conversion of saccharides into FUR and HMF, at 170 ºC. The substrates used were xylose, fructose and glucose as typical monosaccharides, sucrose and cellobiose as examples of disaccharides, and xylan (a polymer composed mainly of xylose) and inulin (a polymer of fructose) as polysaccharides.

## 2. Results and Discussion

### 2.1. Synthesis and characterisation of the catalyst

**Figure 1:**
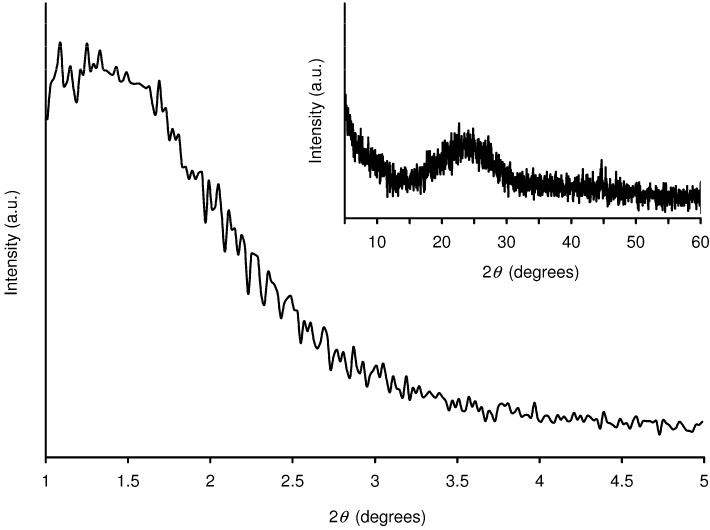
Powder XRD pattern of Al-TUD-1.

Al-TUD-1 was prepared as described previously using aluminium(III) isopropoxide and tetraethylorthosilicate as the Al and Si sources, respectively, and triethanolamine as the template [20]. After calcination, elemental analysis indicated a Si/Al ratio of 21, which is close to the ratio of 25 used in the synthesis gel. The powder X-ray diffraction (XRD) pattern of Al-TUD-1 shows only one broad peak at low angle (*ca.* 1.4º 2*θ*) and a very broad peak centred around 24º 2*θ* (Figure 1), indicating that the material is amorphous, but has the characteristics of a mesostructured material [19,21,24,25]. No evidence of crystalline alumina or other phases can be detected in the pattern.

**Figure 2:**
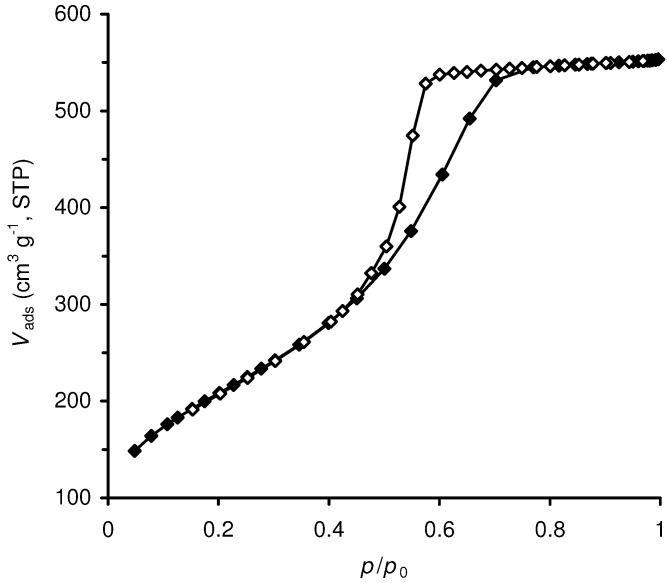
N_2_ adsorption-desorption isotherms at -196 ºC of Al-TUD-1.

Al-TUD-1 exhibits a type IV N_2_ adsorption isotherm with a H2 hysteresis loop (Figure 2), which is consistent with the presence of a disordered mesoporous material with an interconnected (worm-like) pore network [22,26,27,28]. 

The capillary condensation in the mesopores occurs in the relative pressure range of about 0.5 to 0.7, above which the adsorption branch levels off and no more adsorption takes place in the higher relative pressure region (external surface area is negligible). Similar results were reported previously for purely siliceous and metal-incorporated TUD-1 samples [19,21,22,29]. 

**Figure 3:**
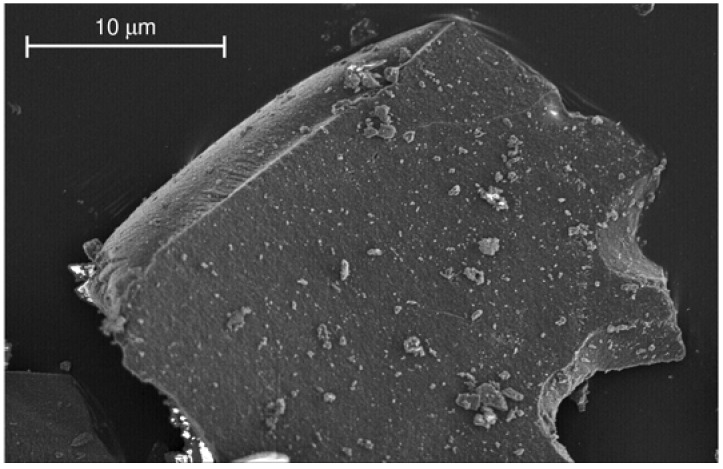
Representative SEM image of Al-TUD-1.

The isotherm for Al-TUD-1 gave a BET specific surface area of 757 m^2^ g^–1^, and average pore diameters of 4.3 nm (calculated from the adsorption branch, using the BJH method) and 3.9 nm (calculated from the desorption branch). SEM analysis of Al-TUD-1 showed particles with an uneven shape and size (Figure 3).

**Figure 4:**
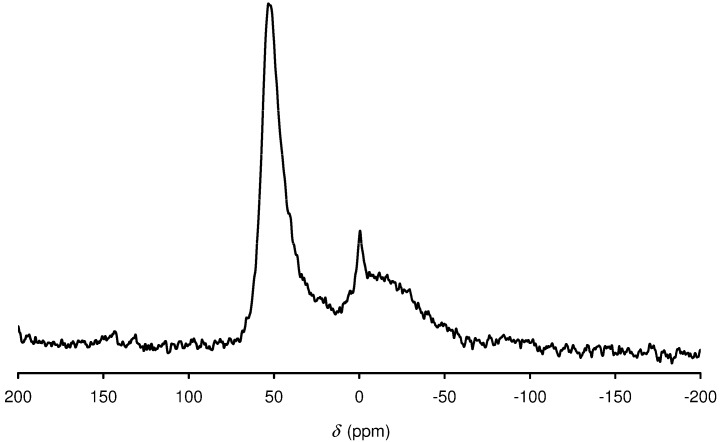
^27^Al MAS NMR spectrum of Al-TUD-1.

The nature of Al in Al-TUD-1 was investigated by using ^27^Al-NMR spectroscopy (Figure 4). The spectrum exhibits a strong resonance at *δ* = 53 ppm, which can be assigned to tetrahedrally coordinated (structural) aluminium species. A high-field signal at *δ* = 0 ppm is attributed to hexacoordinate Al centres. The spectrum is very similar to that described previously for Al-TUD-1 with Si/Al = 30 prepared using triethanolamine as the template [30].

The Brønsted (B) and Lewis (L) acid properties of Al-TUD-1 were quantified by adsorption of pyridine followed by FTIR. Pyridine was chosen as the basic probe since its critical dimension of *ca.* 0.65 nm [31] is comparable with the size of the xylose molecule. The sample showed both L and B sites interacting with pyridine after outgassing at 150 ºC: [B] = 59 μmol g^–1^, [L] = 138 μmol g^–1^. Although the L sites are probably due to defective framework and/or extra-framework Al species, we cannot exclude the possibility that L sites may also arise from tetrahedral aluminium. Indeed, in a study of the adsorption of pyridine on mesoporous aluminosilicate SBA-15 molecular sieves, Luan and Fournier reported that the tetrahedral aluminium centres contributed only to Lewis acidity [32]. Normally, in crystalline zeolite materials, tetrahedral aluminium is expected to form bridging hydroxyl groups (Si–OH–Al), contributing to Brønsted acidity. Luan and Fournier reasoned that tetrahedral aluminium in mesoporous aluminosilicates with amorphous pore walls could contribute to Lewis acidity due to crystallographic disorder at the atomic level. For Al-TUD-1, at 350 ºC pyridine desorbed more easily from the B sites than from the L sites. Thus, the ratio moderate + strong to total Brønsted sites ([B]_350_/[B]_150_) was 0.02, indicating that most of the sites are of a rather weak nature, while the Lewis ratio ([L]_350_/[L]_150_) was 0.61. Nevertheless, it is worth mentioning that the number of Brønsted sites in Al-TUD-1 may be underestimated since pyridine is a weak base (compared with, for example, ammonia) and may not be able to deprotonate the weaker sites present in the sample (nevertheless, ammonia may lead to an overestimate of the effective number of acid sites because of its smaller molecular dimensions in comparison to the saccharide molecules) [33].

### 2.2. Catalysis

#### 2.2.1. General considerations

**Figure 5:**
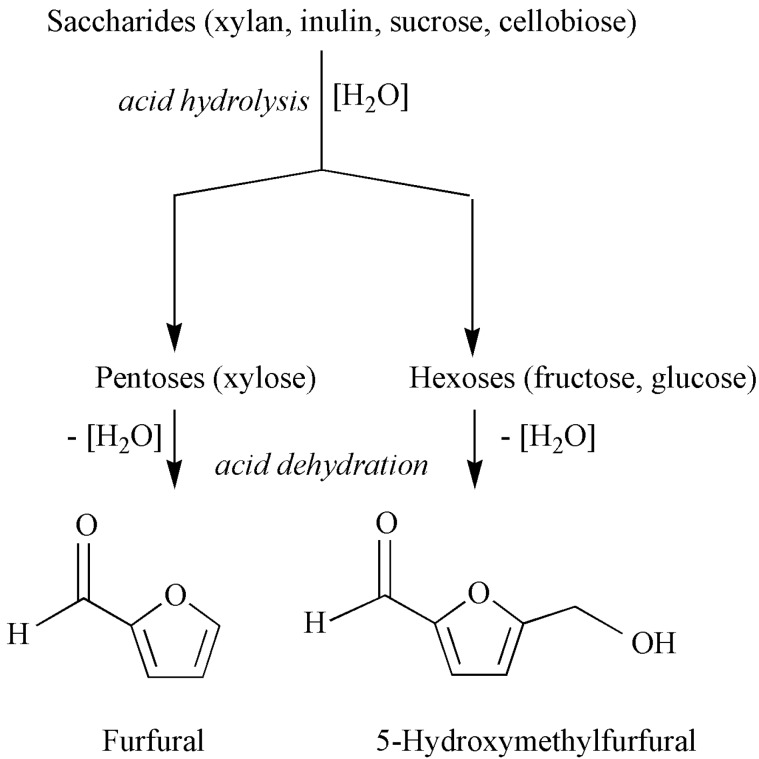
Simplified representation of the acid hydrolysis and dehydration of saccharides into FUR and HMF.

The liquid phase conversion of saccharides under nitrogen in the presence of Al-TUD-1 was investigated at 170 ºC using a water-toluene biphasic solvent system. Unless otherwise specified, product yields are reported in mol %. FUR and HMF selectivities are improved by using an organic extracting solvent since the reaction of the saccharides takes place in the aqueous phase, and the product FUR or HMF is partially transferred into the organic phase [34,35]. The acid hydrolysis of di/polysaccharides gives monosaccharides; dehydration of pentoses and hexoses gives FUR and HMF, respectively, via the elimination of three water molecules per molecule of monosaccharide (Figure 5) [5,36,37]. 

**Figure 6:**
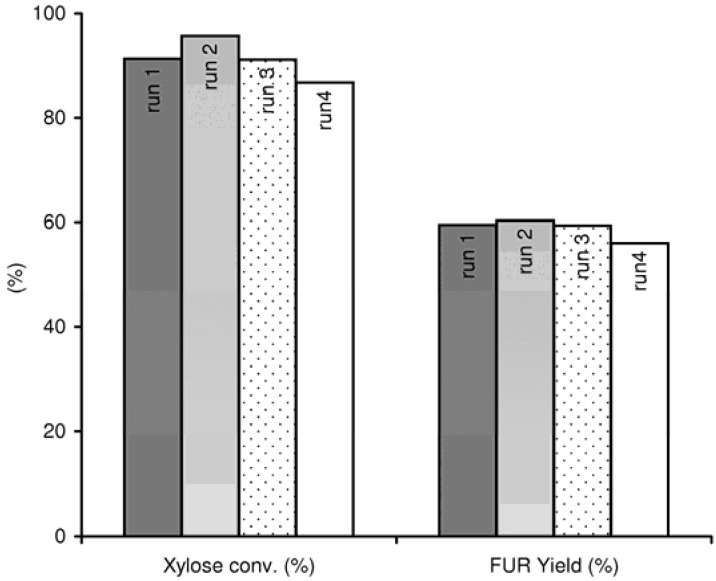
Reaction of xylose in the presence of Al-TUD-1 at 170 ºC (four consecutive 6 h batches).

Catalyst stability tests were carried out for Al-TUD-1 by performing four consecutive 6 h batches of the xylose reaction. After each run, the catalyst was separated, washed with methanol, and activated at 350 ºC for 3 h with a heating rate of 1 ºC min^–1^. Conversions of xylose (87–96%) and FUR yields (56–60%) in recycling runs were quite similar (Figure 6). The Si/Al mole ratio of the recovered solid was 22, which is comparable to that of the fresh catalyst (experimental error of the ICP-OES analyses for Al and Si is 6.0 and 7.2%, respectively), suggesting that Al-TUD-1 is fairly stable under the reaction conditions used.

#### 2.2.2. Pentose-based carbohydrate feedstock

**Figure 7:**
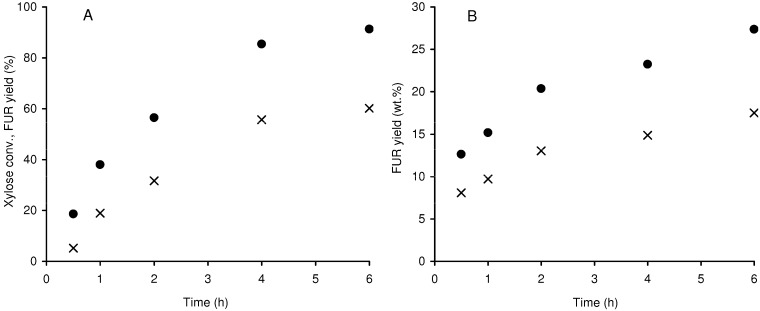
Reaction of xylose (A: (●) conversion; (×) FUR yield), and reaction of xylan (B: (●) xylose yield; (×) FUR yield), in the presence of Al-TUD-1 at 170 ºC.

The reaction of xylose in the presence of Al-TUD-1 gave 56%/60% FUR yield at 4 h/6 h (Figure 7A). These results compare favourably with those reported for a delaminated zeolite (Si/Al = 29) obtained by swelling and ultrasonication of a layered precursor of Nu-6(2) (46% FUR yield [38]), H-mordenite zeolite (Si/Al ~ 6, 34% at 4 h [38]), and microporous silicoaluminophosphates (FUR yields of 41–48% for SAPO-11 at 6 h [11]), used as solid acid catalysts in the same reaction, under similar conditions.

The conversion of xylose is 91% at 6 h, indicating that by-products amount to 31% yield. During the reaction of xylose the initially colourless water/toluene phases turned yellow-orange and the white powdered catalyst turned brown, suggesting the presence of organic by-products. However, significant amounts of organic by-products were not detected by chromatographic techniques (HPLC of the aqueous phase and GC-MS of the toluene phase). Possibly, soluble oligo/polymeric products are formed. According to the literature, by-products may be formed via fragmentation and/or condensation reactions involving intermediates and FUR [5,39]. The one-pot conversion (hydrolysis and dehydration) of xylan in the presence of Al-TUD-1 gave xylose (hydrolysis product) and FUR in increasing amounts reaching 27 and 18 wt % yield at 6 h (Figure 7B); the theoretical FUR yield is ca. 73 wt %.

#### 2.2.3. Hexose-based carbohydrate feedstock

**Figure 8:**
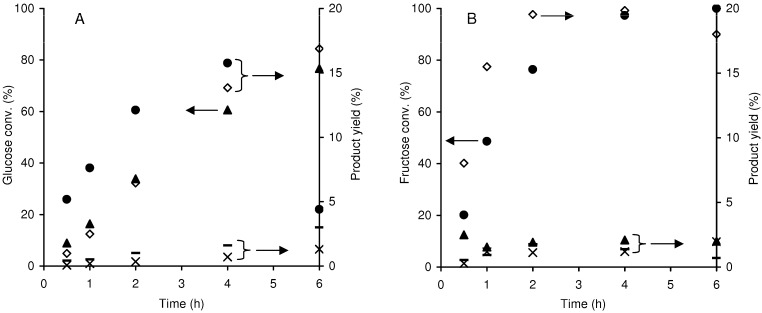
Reaction of glucose (A: (▲) conversion; (●) fructose yield), and reaction of fructose (B: (●) conversion; (▲) glucose yield), in the presence of Al-TUD-1 at 170 ºC. The HMF (□), (–) mannose and FUR (×) yields are also shown.

Figure 8 shows the catalytic results obtained for the reactions of fructose and glucose in the presence of Al-TUD-1, at 170 ºC. For both hexoses, the isomers glucose, fructose and mannose were simultaneously present, with the mannose yield always being less than 3%. With fructose as the substrate, glucose was formed in less than 3% yield, and with glucose as the substrate, fructose yield reached a maximum of ca. 16% at 61% conversion. These results suggest that, under the applied reaction conditions, the isomerisation of glucose to fructose is more important than the reverse reaction, in agreement with the literature [34,40]. Based on the kinetic profiles, the reactivity of fructose is higher than that of glucose, and fructose gives somewhat higher HMF yields, at least until *ca.* 75% conversion. In the case of glucose, HMF may be formed from fructose (the isomerisation product), and this pathway may be in competition with major glucose degradation pathways [40,41,42]. Similar to that observed for xylose, for the hexoses, colour build-up was observed for the liquid phases and the catalyst, although no significant amounts of by-products were detected. FUR was always detected in minor amounts (< 2% yield at 6 h reaction of hexose), and may be formed via consecutive tautomerisation and retro-aldol reactions [40,42]. Levulinic acid (a possible product of the hydrolysis of HMF) was never detected, possibly because the acid sites of Al-TUD-1 are not strong enough: more acidic conditions are required for converting HMF into levulinic acid than for HMF formation [43]. According to the literature, HMF may be involved in the formation of coke deposits on the microporous surface of aluminosilicates [44]. Taking into consideration that polymerization reactions may be enhanced under relatively weak acidic conditions [43], and that Al-TUD-1 possesses mainly Lewis acid sites and weak Brønsted acidity, it is possible that by-products are essentially soluble polymers.

Somewhat in parallel with those observed for Al-TUD-1, the published results for microporous zeolites used as solid acids in the conversion of hexoses into HMF seem better for fructose than for glucose as substrate. The reaction of glucose in the presence of zeolite H-Y (Si/Al = 6.5) gave less than 10% HMF yield at *ca.* 75% conversion, at 160 ºC [44]. In contrast, the reaction of fructose in the presence of H-beta, H-ZSM-5, H-mordenite and H-faujasite zeolites with Si/Al ratios in the range of 10–100 was quite selective towards HMF, with one of the best results being 91% HMF selectivity at 76% conversion [8,44]. The Si/Al ratio influenced the rate of the reaction of fructose into HMF, most likely due to changes in the acid properties and/or the surface polarity of the catalyst. 

**Figure 9:**
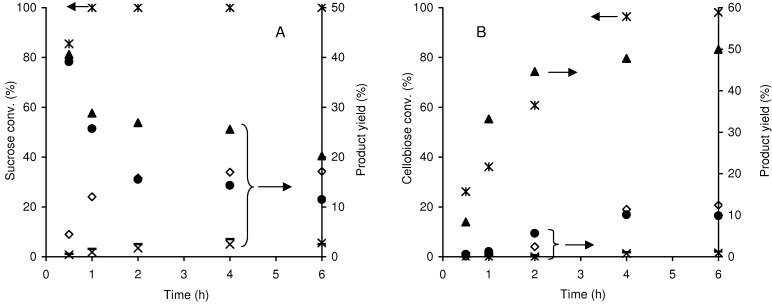
Reaction of sucrose (A) and of cellobiose (B), in the presence of Al-TUD-1 at 170 ºC: (*) disaccharide conversion; (●) fructose yield, (▲) glucose yield; (□) HMF yield; (–) mannose yield; (×) FUR yield.

The one-pot conversion of the disaccharides sucrose [glucose and fructose linked by a β(1→2) glycosidic bond] and cellobiose [two glucose units linked by a β(1→4) glycosidic bond] in the presence of Al-TUD-1 gave 100% conversion at 1 h reaction and 98% conversion at 6 h, respectively, indicating that sucrose is more reactive than cellobiose (Figure 9). A similar relation of substrate reactivity may be established for zeolite H-Y (Si/Al = 15) used as acid catalyst in the same reactions (monosaccharide yield >88%) [45,46]; the Si/Al ratio may have an important effect on the hydrolysis reaction rate [47]. The reaction of sucrose in the presence of Al-TUD-1 gave glucose and fructose as the main products at 30 min reaction (formed in ca. 40% yield each), indicating that relatively fast hydrolysis takes place (Figure 9A). After 30 min reaction, the monosaccharide yields decrease, especially for fructose (consistent with the fructose reactivity being higher than that for glucose, referred to above), and a maximum HMF yield of 17% was reached. In the case of cellobiose, glucose is the main product at 6 h reaction (50% yield), fructose yield is 10%, and HMF yield is 12% (Figure 9B). For both disaccharides minor amounts of mannose and FUR were formed during the 6 h reaction (as observed with fructose and glucose as feedstocks). The undesirable reaction pathways may be similar to those occurring for fructose and glucose (give a maximum HMF yield of 20% and 17%, respectively). 

**Figure 10:**
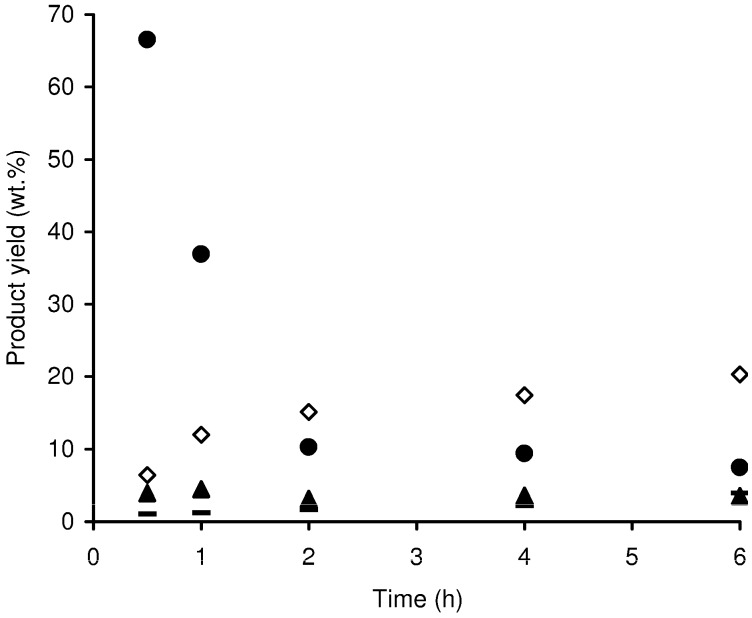
Reaction of inulin in the presence of Al-TUD-1 at 170 ºC: (●) fructose yield; (▲) glucose yield; (–) mannose yield; (□) HMF yield.

The one-pot conversion of inulin (a fructan, used in 10 wt %) in the presence of Al-TUD-1 gave mainly fructose at 30 min reaction (67 wt % yield), Figure 10. 

A fast drop in fructose yield with time was accompanied by the formation of HMF, reaching 20 wt % yield at 6 h (the theoretical yield is approximately 78 wt %). Glucose and mannose were minor products formed via reversible isomerisation (< 5 wt % yield). The selective hydrolysis of inulin (avoiding HMF formation) has been successfully carried out using H-Y zeolite at lower reaction temperatures (ca. 92% fructose yield, at 90 ºC) [46].

## 3. Experimental

### 3.1. General

All reagents, substrates, solvents and standards were obtained from commercial sources and used as received: aluminum(III) isopropoxide (≥99%), D-(+)-cellobiose (≥99%), inulin, D-(+)-sucrose (≥99%) and phenol (≥99.5%) from Fluka; isopropanol (99%), ethanol (99%), triethanolamine (99.9%), tetraethylammonium hydroxide (99.9%), D-(-)-fructose (>99%) and D-(-)-ribose (98%) from Aldrich; tetraethylorthosilicate (99.9%) and 4-O-methyl-D-glucurono-D-xylan from Sigma; D-(+)-glucose (>99%), D-xylose (99%) and toluene (≥99.9%) from Sigma-Aldrich; D-(-)-mannitol (>99%) from Riedel-de Haën.

ICP-AES measurements for Si and Al were carried out at the Central Laboratory for Analysis, University of Aveiro (by E. Soares and co-workers). The ^27^Al magic-angle spinning (MAS) NMR spectrum was measured at 104.26 MHz with a Bruker Avance 400 (9.4 T) spectrometer, using a contact time of 0.6 μs, a recycle delay of 0.8 s, and a spinning rate of 15 kHz. Chemical shifts are quoted in ppm from Al(H_2_O)_6_^3+^. Powder X-ray diffraction (XRD) data were collected at room temperature on a Philips X’Pert MPD diffractometer, equipped with an X’Celerator detector, a graphite monochromator (Cu-Kα X-radiation, *λ* = 1.54060 Å) and a flat-plate sample holder, in a Bragg-Brentano para-focusing optics configuration (40 kV, 50 mA). Samples were step-scanned in 0.04º 2*θ* steps with a counting time of 6 s per step. SEM was carried out on a Hitachi SU-70 UHR Schottky instrument. The BET specific surface area was estimated from N_2_ adsorption isotherm measured at -196 ºC using a Micromeritics ASAP 2010 instrument: the sample was outgassed at 350 ºC under vacuum. The acid properties were measured using a Nexus-Thermo Nicolet FTIR instrument (64 scans and resolution of 4 cm^–1^) equipped with a specially designed cell, using self-supported discs (5–10 mg cm^–2^) and pyridine as the basic probe molecule. After *in situ* outgassing at 450 ºC for 3 h (10^–6^ mbar), pyridine (99.99%) was contacted with the sample at 150 ºC for 10 min and then evacuated at 150 and 350 ºC (30 min) under vacuum (10^–6^ mbar). The IR bands at *ca.* 1540 and 1455 cm^–1^ are related to pyridine adsorbed on Brønsted (B) and Lewis (L) acid sites, respectively [33]. Thermogravimetric analysis (TGA) and differential scanning calorimetry (DSC) were carried out under air using Shimadzu TGA-50 and DSC-50 systems.

### 3.2. Synthesis of Al-TUD-1

Al-TUD-1 was prepared as described previously [20]. Briefly, tetraethylorthosilicate (17.3 g, 83.0 mmol) was added to aluminium(III) isopropoxide (0.68 g, 3.33 mmol) dissolved in a mixture of isopropanol (6.5 mL) and ethanol (6.5 mL). After stirring for a few minutes, a mixture of triethanolamine (12.51 g, 83.9 mmol) and water (9.4 g) was added, followed by addition of tetraethylammonium hydroxide (35 wt % in water, 11.12 mL, 27.0 mmol) under vigorous stirring. The clear gel obtained was stirred at room temperature for 24 h and dried at 98 ºC for 24 h, followed by hydrothermal treatment in a Teflon-lined stainless steel autoclave at 180 ºC for 8 h. Finally, the solid was calcined at 600 ºC in static air for 10 h with a temperature ramp of 1 ºC min^–1^.

### 3.3. Catalytic experiments

Batch catalytic experiments were performed under nitrogen in a tubular glass micro-reactor equipped with a valve for gas purging. In a typical procedure, mono/disaccharide (30 mg) or polysaccharide (10 mg), powdered catalyst (20 mg), H_2_O (0.3 mL) and toluene (0.7 mL) were poured into the reactor. The reaction mixtures were stirred magnetically at 700 rpm and heated with a thermostatically controlled oil bath. Zero time was taken to be the instant the micro-reactor was immersed in the oil bath: individual experiments were performed for a given reaction time. The products present in the aqueous phase were analysed using a Knauer K-1001 HPLC pump, coupled to a Knauer 2300 differential refractive index detector (for sugars) and a Knauer 2600 UV detector (280 nm, for FUR and HMF). For pentose-based feedstocks a PL Hi-Plex H 300 mm × 7.7 mm (i.d.) ion exchange column (Polymer Laboratories Ltd., UK) was used: the mobile phase was 0.01 M H_2_SO_4_; flow rate 0.6 mL min^–1^; column temperature 65 ºC. In the case of hexose-based feedstocks, a PL Hi-Plex Ca 300 mm × 7.7 mm (i.d.) ion exchange column (Polymer Laboratories Ltd., UK) was used: the mobile phase was freshly prepared distilled and deionized water; flow rate 0.5 mL min^–1^; column temperature 80 ºC. The products present in the organic phase were quantified using a Gilson 306 HPLC pump and a Spherisorb ODS S10 C18 column, coupled to a Gilson 118 UV-vis detector (280 nm). The mobile phase consisted of 30% (v/v) methanol in an aqueous solution with 10% methanol (flow rate 0.7 mL min^–1^). Authentic samples of reagents and products were used as standards for measuring calibration curves. Conversion (%) was calculated as 100×(moles of substrate consumed)/(initial moles of substrate). For monosaccharides, FUR or HMF yields (mol %) were calculated as 100×(moles FUR or HMF formed)/(initial moles of monosaccharide). For disaccharides, HMF or monosaccharide yields (mol %) were calculated as 100×(moles of product formed)/(2×(initial moles of disaccharide)). In the case of the polysaccharides, the FUR or HMF yields (wt %) were calculated as 100×(mass of product formed)/(initial mass of polysaccharide). In order to check the data, the experiments were carried out in duplicate and the mean values were calculated.

## 4. Conclusions

The reaction of pentoses (xylose) and hexoses (glucose and fructose) in the presence of Al-TUD-1, at 170 ºC, gave FUR and HMF, respectively; the reactions of hexoses gave 17–20% HMF yields at 6 h, and the reaction of xylose gave 60% FUR yield. Possibly, the acid properties of Al-TUD-1 (mainly Lewis acid sites and weak Brønsted acidity) are more favourable for converting xylose into FUR than hexoses into HMF. The one-pot hydrolysis/dehydration of sucrose and cellobiose in the presence of Al-TUD-1 gave the monosaccharides via the hydrolysis of the glycosidic bonds, which are subsequently dehydrated into HMF, obtained in 17% and 12% yield from sucrose and cellobiose, respectively. In the case of the polysaccharides, xylan and inulin, FUR and HMF were formed in 18 and 20 wt % yield at 6 h, respectively. Based on the colour build-up of the solvents (colourless-yellow-orange) and of the catalyst (white-brown-black) during the course of the reactions, and the fact that no significant amounts of by-products were detected, it is postulated that the formation of soluble polymers and coke deposits may be important competitive reactions, affecting FUR and HMF yields.

Based on the catalytic results for four consecutive 6 h batches carried out for the reaction of xylose, Al-TUD-1 seems to be a fairly stable catalyst. FUR yields at 6 h were in the range 56-60%. The catalytic performance of Al-TUD-1 may be improved by fine-tuning the acid properties (varying the Si/Al ratio [49]) and optimising the reaction conditions.
